# Learning to Diagnose Cirrhosis with Liver Capsule Guided Ultrasound Image Classification

**DOI:** 10.3390/s17010149

**Published:** 2017-01-13

**Authors:** Xiang Liu, Jia Lin Song, Shuo Hong Wang, Jing Wen Zhao, Yan Qiu Chen

**Affiliations:** 1 School of Computer Science, Shanghai Key Laboratory of Intelligent Information Processing, Fudan University, Shanghai 201203, China; xiangliu09@fudan.edu.cn (X.L.); sh_wang@fudan.edu.cn (S.H.W.); jingwenzhao13@fudan.edu.cn (J.W.Z.); 2 School of Electronic and Electrical Engineering, Shanghai University of Engineering Science, Shanghai 201620, China; 3 Department of ultrasound, Changzheng Hospital Affiliated to Second Military Medical University, Shanghai 200003, China; jialin19810818@126.com

**Keywords:** ultrasound imaging, computer-aided diagnosis, cirrhosis, convolutional neural network

## Abstract

This paper proposes a computer-aided cirrhosis diagnosis system to diagnose cirrhosis based on ultrasound images. We first propose a method to extract a liver capsule on an ultrasound image, then, based on the extracted liver capsule, we fine-tune a deep convolutional neural network (CNN) model to extract features from the image patches cropped around the liver capsules. Finally, a trained support vector machine (SVM) classifier is applied to classify the sample into normal or abnormal cases. Experimental results show that the proposed method can effectively extract the liver capsules and accurately classify the ultrasound images.

## 1. Introduction

Hepatic cirrhosis is a chronic, degenerative disease in which normal liver cells are damaged and are then replaced by scar tissue [1]. It changes the structure of the liver and the blood vessels that nourish it. The disease reduces the ability of liver to synthesise proteins and produce hormones, nutrients, medications and poisons. Chronic liver infection such as hepatitis B is the primary cause of cirrhosis. Other causes include long-term alcoholism, drugs or toxins, certain parasitic infections, and so on. Ongoing liver damage with liver cell degeneration and necrosis repeatedly followed by fibrous tissue hyperplasia and hepatocyte regeneration results in cirrhosis. The main signs and symptoms of cirrhosis are the damage of liver function and portal hypertension and frequently related to the complications such as esophageal and gastric variceal bleeding, hepatic encephalopathy, secondary infections, and so on. Hepatic cirrhosis is one of the common causes of death reported by WHO (World Health Organization).

**Figure 1:**
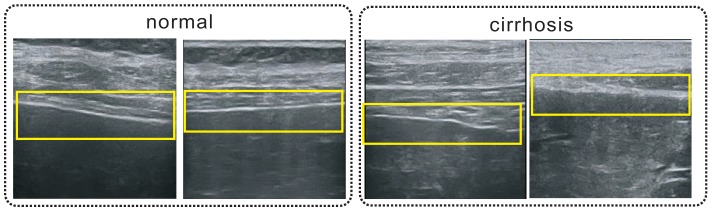
Different manifestations on liver capsules (inside the yellow boxes) between normal and diseased samples.

Ultrasound, CT and MRI are routinely used in the diagnosis of cirrhosis. Medical ultrasound imaging as a non-invasive and non-ionizing technique has been widely used by doctors to diagnose cirrhosis. Computer-aided diagnosis systems can be used to diagnose cirrhosis at an early stage based on ultrasound images for timely treatments [2]. B-scan ultrasound imaging produces a specific texture pattern which reflects the spatial distribution of the tissue scatterers relative to the wavelength of the incident pulse. The obtained B-scan ultrasound image appears as a granular texture [3]. High frequency ultrasound imaging can obtain the superficial section images of the liver with higher resolution that reflect more subtle changes in the liver capsule and parenchyma at early stage of cirrhosis, which is better than the traditional convex array ultrasound probe [4]. For a normal liver, the parenchyma has homogeneous echotexture, and the liver capsule is smooth with even thickness. The progression of cirrhosis will result in damaged parenchyma tissue, fibrosis and formation of pseudolobule. The liver capsule will be manifested as thickening or have uneven thickness, with wavy, jagged, and stepped changes as shown in Figure 1.

**Figure 2:**
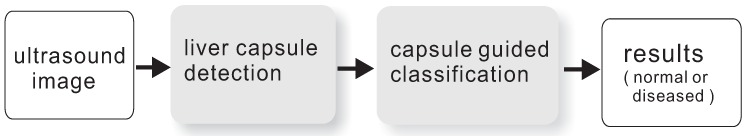
The flowchart of the proposed method.

Computer-aided cirrhosis diagnosis systems can contribute to early diagnosis in order to implement target treatment, which can save more people. However, from a technical perspective, automatic diagnosis of cirrhosis from ultrasound images is a challenging task due to the highly complex structure of the liver. We propose in this paper a system that can solve this problem effectively. The flowchart of the proposed method is shown in Figure 2. We first propose a method which is able to localize the liver capsules in the ultrasound image, which is the key building block of our approach. The geometrical property of the extracted liver capsule curves can serve as a preliminary indicator of cirrhosis. Since the tissues above and below the liver capsules are significantly different, liver capsule can also serve a guideline for the image classification task. With the guidance of the liver capsule, we can extract features precisely from the patches from the tissues on, above and below the liver capsule. By introducing a transferred deep classification model into this framework, our method has achieved remarkable performance in binary classification task (cirrhosis or not) of the ultrasound images.

## 2. Related Works

Clinicians diagnose cirrhosis and determine its progression stages based on the above observations. However, human diagnosis depends on a large amount of training and experience. Sometimes, misdiagnosis due to subjective factors may have serious consequences. Hence, computer-aided diagnosis systems based on quantitative analysis are of great value [5,6].

Existing computer-aided cirrhosis diagnosis systems mainly focus on quantitatively analyzing the texture of parenchyma in liver ultrasound images by extracting texture features such as fractal features [7], statistical texture features [8,9,10], spectral features [11,12,13,14] or combined features [2,15,16,17,18]. Then different classifiers such as random forest, support vector machine, neural networks, etc., are trained to classify the samples into cirrhosis or normal ones.

Virmani et al. [14] applied multiresolution wavelet packet transform (WPT) to extract the parenchyma texture. Then Genetic algorithm-SVM was employed to select features and classify the sample image into normal, cirrhosis and HCC liver. Lee et al. [13] combined texture features extracted by M-band wavelet transform and Gabor wavelet to obtain features of the parenchyma and applied ensembled classifiers to classify the image samples into different liver diseases or normal ones. However, such texture features do not directly correspond to the nodular fibrotic of liver parenchyma, which is not consistent with the visual attributes for medical diagnosis. Wu et al. [2] applied roughness and granularity texture descriptors derived from Laws’ masks, gray level co-occurrence matrix, Fourier power spectrum and gray level difference statistics to classify samples into normal, hepatoma or cirrhosis ones. However, image noises and other tissues included in parenchyma region that may destroy the homogeneous structure may result in failure of the method. Lee et al.’s method [7] constructs the parenchyma texture feature based on the fractal dimension and M-band wavelet transform. Different classifiers were tried and the highest correct rate was obtained with back propagation neural network. These fractal dimension based method achieved satisfying performance in distinguishing normal and abnormal liver, but better methods are needed to further determine the cirrhosis stages. Several proposals combine several feature extraction methods together [2,15,16,17,18], which make use of the advantages of different features. However, the performance relies on a good feature selection method such as genetic algorithm [14,19], singular value decomposition [9], particle swarm optimization [17], etc.

To sum up, the features of liver capsule are not well investigated because liver capsule is also an important diagnostic criterion for cirrhosis diagnosis. Early methods that investigate liver capsule [4,20] diagnose cirrhosis by analyzing the features of capsule qualitatively. However, these qualitative methods are subjective and the diagnostic results are not reliable [20]. Liu et al.’s method [21] quantitatively analyzes the liver capsule and proposed three features to describe the liver capsule. However, their method relies on manual initialization.

## 3. Liver Capsule Detection

The proposed liver capsule detection framework consists of two stages: sliding window detection and dynamic programming based linking. Sliding window detector performs per-pixel classification and is able to generate a detection response map. Then, from the response map, a complete liver capsule curve can be extracted through a dynamic programming based linking method.

### 3.1. Sliding Window Detector

**Figure 3:**
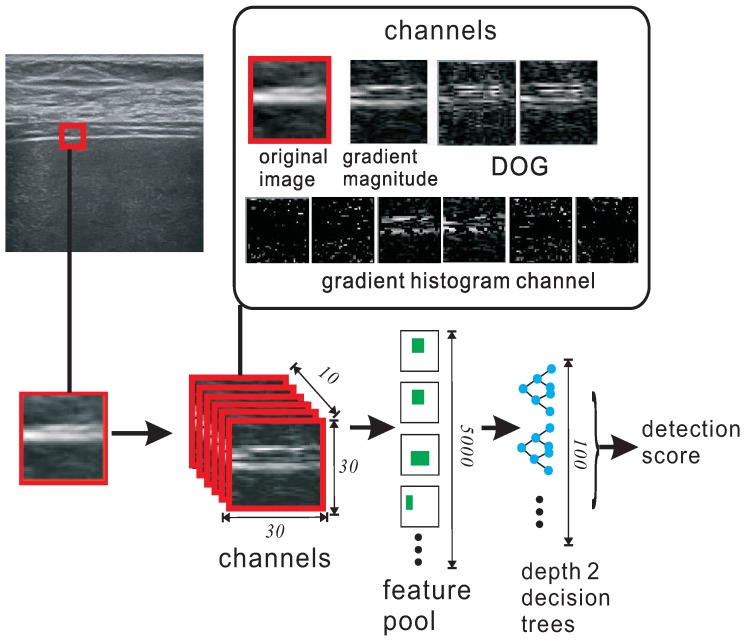
The pipeline of sliding window detector.

Automatic detection of the liver capsule is a challenging task, since there are many similar structures in the image. We adopt the sliding window detection framework in which a classifier is trained to classify the image patch in a sliding window. The key issue in this framework is how to extract a set of features that are discriminative enough. Inspired by the work in [22], we develop a method which learns the most discriminative features from multiple distinctive channels. These channels are transformed from the original image with various linear and non-linear filters. Then, an over-complete set of rectangular features which we call feature pool are extracted from these image channels as shown in Figure 3. Afterwards, a subset of most discriminative features are selected from the feature pool with AdaBoost.

Consider a set of training sample $\left(f_{1},c_{1}\right),\left(f_{2},c_{2}\right),...,\left(f_{m},c_{m}\right)$, where $f_{i}$ is a $N_{f}$ dimensional feature vector, and $c_{i}$ is its class label (positive or negative). AdaBoost associates each training sample a weight $w_{i}$ which equals $\frac{1}{N_{f}}$ initially. Then the following two steps are repeated *T* times:

1. Train a depth-two decision tree $h(f_{i})$ which minimizes the weighted classification error: $\varepsilon_{t}=\sum_{i}w_{t}^{i}\left|h\left(f_{i}\right)-c_{i}\right|$, where *t* is current number of iterations.
2. Update the weight according to: $w_{t+1}^{i}=w_{t}^{i}\beta_{t}^{1-e_{i}}$, where $e_{i}$ is 1 if $f_{i}$ is correctly classified and it equals to 0 otherwise, and $\beta =\frac{\varepsilon_{t}}{1-\varepsilon_{t}}$.

A depth-two decision tree consists of a root node and two leaf nodes. Each node of the depth-two decision tree consists of a feature index *j*, a threshold *θ* and a polarity *p*(+1 or −1). If $pf_{i}\left(j\right)>p\theta$ then $f_{i}$ is passed down to the left child. Otherwise, it is passed down to the right child. The tree is trained greedily: the root node is first determined by selecting (j,θ,p) that minimizes $\varepsilon_{t}$, then the training data is split into two parts, the left and right leaf node are then trained in the same way with the two parts of data, respectively.

We use four kinds of channels: original image, gradient magnitude, gradient histograms, difference of Gaussian (DOG) (shown in Figure 3). $N_{f}=5000$ rectangular features are selected with random positions and sizes from randomly picked channels. Then these features are selected with adaboost, we use a depth-two decision tree as the weak classifier, since it can better explore the relationship between features. We use conventional discrete boost and T=100 weak classifiers $\left\{h_{i}\right\}$ are selected. Each weak classifier is a depth-two decision tree which consists of three binary tests with three features [22]. The final detection score at location (x,y) is computed by voting the results of the weak classifiers:

(1) $$R\left(x,y\right)=\sum_{i=1}^{T}h_{i}\left(f\left(x,y\right)\right).$$

The size of each sliding window is 30 by 30 pixels. Detection is performed in a single scale, an image can be processed in less than one second.

Compared with a conventional VJ detection framework [23], the above integrated channel feature framework has several advantages: firstly, it is able to leverage information from multiple heterogeneous sources such as gradient, DOG, etc. Secondly, the detector can be trained much faster (within several minutes), while the VJ detector may take several days to train.

### 3.2. Linking by Dynamic Programming

Now we have trained a sliding window detector which is able to perform per-pixel classification to find pixels that are likely to belong to liver capsule, but there are still false positives in the detection response. The detector can only generate a response map rather than a complete liver capsule curve. In order to further extract a complete curve from the detection response, we propose a dynamic programming algorithm which can link the detections that are most likely to form the liver capsule. We formulate the problem as finding a curve that goes from the left boundary to the right boundary of the image with the highest summed score. This can be achieved by a dynamic programming algorithm in which the cost of a location (x,y) can be computed recursively as:

(2) S(x,y)=max(S(x−1,y−1),S(x−1,y),S(x−1,y+1))+R(x,y)

**Figure 4:**
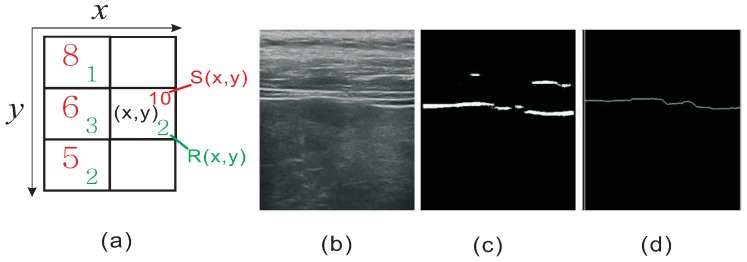
(**a**) An example of how S(x,y) is computed; (**b**) Original image; (**c**) Response map of the detector; (**d**) The curve extracted after dynamic programming based linking.

An example is given in Figure 4a, the summed score at the middle-right pixel (x,y) is computed as the sum of maximum values of left pixels and R(x,y) (8+2=10). When computing the score, each pixel location (x,y) preserves the precedent pixel location to the left. A path with the maximum score can be determined by firstly finding the pixel on the right side of the image with the maximum *s*. Since each pixel preserves the precedent one, a complete path can be determined by backtracking the pixel locations. By finding the path with the maximum score, a complete liver capsule which effectively links the fragmented detections and filters out false positive detections can be extracted. One sample of liver capsule detection is shown in Figure 4.

## 4. Liver Capsule Guided Image Classification

**Figure 5:**
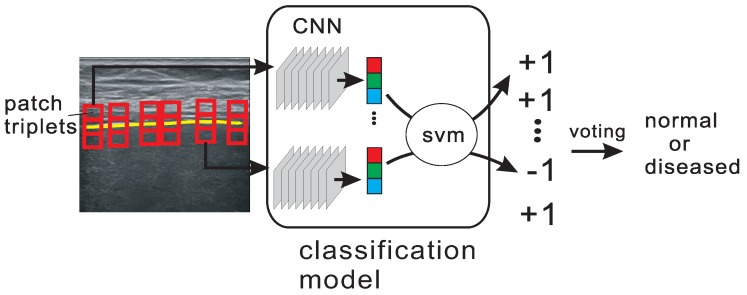
The pipeline of the liver capsule guided image classification.

Our motivation of using a liver capsule to guide the image classification comes from the observations that as cirrhosis progresses, the tissues inside and outside the liver capsule change, and the liver capsule itself may become thicker and diffusive. Based on the above two observations we propose a novel image classification framework in which features are extracted from a set of image patch triplets. Given an extracted liver capsule, a fixed number of points is picked uniformly on the curve. Then for each selected point, three image patches are selected in the way shown in Figure 5. We call these three patches a patch triplet. By extracting features from the patch triplets, we are able to describe the appearance of the liver capsule as well as the tissue structures above and below the liver capsule. A classification model is then trained to classify each patch triplet, and the classification results are collected to make the final decision of classification. The classification of patch triplet may sometimes be inaccurate due to image noise or inaccuracy in liver capsule detection, but as all the patch triplets are combined, the final decision can be expected to be correct with a high probability.

### 4.1. Deep Classification Model with Transfer Learning

The Convolutional Neural Network (CNN) has shown superior performance in many image classification tasks ever since its success in the ImageNet Challenge [24]. Compared with traditional neural networks, CNN has better generalization ability thanks to its delicate local connectivity and weight sharing mechanisms. However, CNN still requires tremendous amount of training data to prevent the model from overfitting. In our problem, it is infeasible to collect a large amount of training samples. Therefore, it is difficult to train a specific CNN model end to end. In fact, we did try to train a CNN model with the data at hand; however, the training process was observed as overfitting since the validation error increases. On the other hand, in the small sample size problem, SVM is preferred due to its excellent generalization power. Our motivation is to leverage the description ability of CNN and the generalization power of SVM.

**Figure 6:**
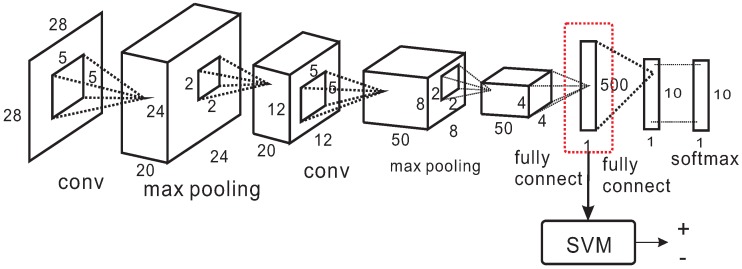
Transferred deep classification model.

It has been proven that learning models trained from one domain can be transferred to another related domain and still be effective. We take advantage of a pretrained 8 layer CNN model trained with hand-written digit data MNIST [25]. The MNIST data consists of 60,000 training samples and 10,000 test samples of hand-written digit images. The network structure is illustrated in Figure 6: two consecutive combinations of convolution layer and max-pooling layer, followed by two fully connected layers and finally a softmax loss layer which generates the probability to each class. Once the network structure is determined, it can be effectively trained using many deep learning libraries. We use the MatConvNet library [26] due to its excellent compatibility with Matlab.

The output of the fully connected layer (before softmax loss layer) is used as the feature of our image classification problem as shown in Figure 6. The features of the three patches are concatenated as one feature vector: $t={\left(p_{1},p_{2},p_{3}\right)}^{T}$. The feature vector then serves as input of SVM, and by training the SVM model, we are able to fine-tune the pretrained CNN model to fit our problem. The final decision of a image *I* is made by combining the SVM classification results of all the *M* patch triplets ${\left\{t_{i}\right\}}_{i=1}^{M}$:

(3) $$F\left(I\right)=\sum_{i=1}^{M}f(t_{i})$$

where f(.) is the classification score output by the trained SVM model.

## 5. Experiments

### 5.1. Performance of the Detector

We evaluate our method on a dataset consisting of 91 ultrasound images in which 44 images are from normal people and 47 images are from people with cirrhosis (The research work in this paper has been approved by the Ethnical Committee of Second Military Medical University in 10 March 2011 (Changzheng Hospital is affiliated to Second Military Medical University)). The patients were diagnosed according to the Child-Pugh score which consists of five clinical measures of the liver disease: Total bilirubin, Serum albumin, Prothrombin time, Ascites and Hepatic encephalopathy. Ultrasound and CT are also referred to for final confirmation. These image are collected with Voluson E8 ultrasound machine (GE Medical Systems) with a high-frequency probe. The frequency of ultrasound was 4–10 MHz. The examinees were in supine or left lateral position. Both the left lobe and right lobe of liver are imaged, and the clearest one is selected by the doctor. Table 1 gives the statistics of the dataset on gender, cirrhosis grade. Since the body shape and thickness of adipose tissue varies among different examinees, the distance and frequency of ultrasound should be adjusted for different examinees. This will result in different pixel sizes with different samples, the mean and standard deviation of the pixel size are 0.0794 mm and 0.0099 mm. We also found the machine output gave different sized images, the image width was 262±36 pixels, and image height was 375±29 pixels.

**Figure 7:**
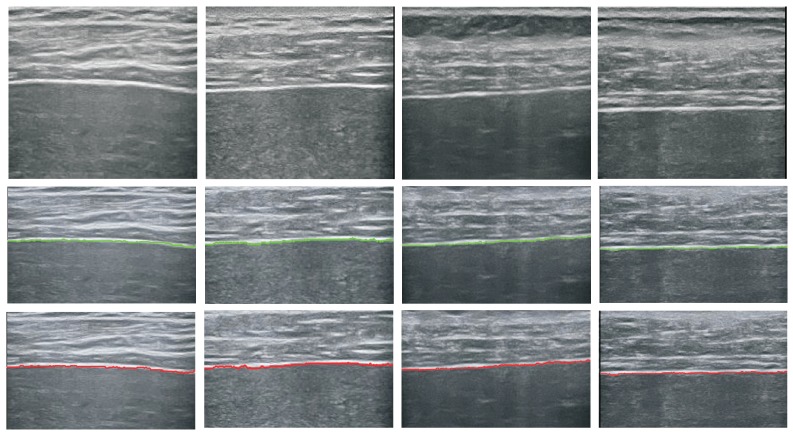
Extracted liver capsules (bottom row) and manually labeled liver capsules (middle row) for normal samples.

**Figure 8:**
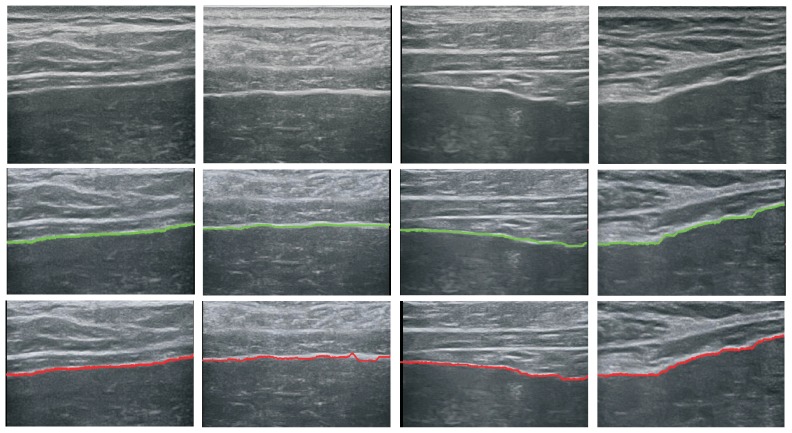
Extracted liver capsules (bottom row) and manually labeled liver capsules (middle row) for diseased samples.

We first evaluate the performance of proposed liver capsule detection method. The liver capsules in all the 91 images were manually labeled as groundtruth to check if the detected curve coincide with the labeled one. Let the extracted curve be a set of sample points: $L=\{\left(x_{i},y_{i}\right)\}$ and the groundtruth curve be $L^{*}=\left\{\left(x_{i}^{*},y_{i}\right)\right\}$, *L* and $L^{*}$ have been aligned and share the same y values which is $y_{i}=y_{\min},y_{\min}+1,...,y_{\max}$. We compute the distance between two sample points as: $d_{i}=\left|x_{i}-x_{i}^{*}\right|$. If the distance is below 20 pixel, we consider *L* and $L^{*}$ coincide at $\left(x_{i},y_{i}\right)$. The completeness of extracted curve can be computed as the percentage of sample points whose $d_{i}$ are below 20 pixels. If $d_{i}$ of 70% of the sample points are below 20 pixel, we consider the detection to be a success. The results are given in Table 2. We can see from the table that our method achieves high performance with normal sample images, this is because the liver capsule in these images are relatively clearer. Several detection results for the normal and the diseased samples as shown in Figure 7 and Figure 8 respectively.

### 5.2. Performance of Image Classification

The performance is evaluated with a 3-fold cross validation scheme: the images are divided into three folds, in each round, two sets are selected as training sample and the rest one as validation samples. The ROC (Price Rate of Change) curves are plotted and the AUC (Area under Curve) is computed and compared to evaluate the performance of the methods. We also report the classification accuracies of the method in the three trials respectively as well as the mean accuracy. In each trial, parameters are tuned with only training data and without using the labels of the test samples.

#### 5.2.1. Impact of Detection Error

**Figure 9:**
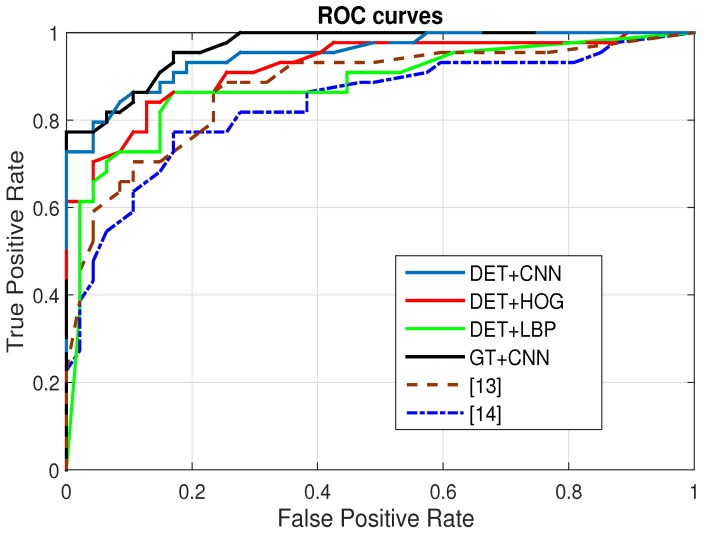
The ROC curves of the proposed method with different low level features.

The proposed liver capsule detection method can be successful in most of the cases but it can also make mistakes. We use the abbreviation DET + CNN to represent our method which means the combination of liver capsule detection and convolutional neural network. In order to understand the impact of the detection errors to the final classification performance, we carry out an experiment which uses the manually labeled liver capsule curves as input to the image classification module. The ROC curve is presented in Figure 9 (GT + CNN) and the AUC is 0.968 as in Table 3 (GT + CNN). Although this is an ideal results, it can serve as an upper-bound for the performance evaluation. From the result, we can understand the performance upper bound of proposed image classification framework.

#### 5.2.2. CNN vs. Hand-Crafted Features

Since our image classification can be equipped with various kinds of low level feature extraction method other than the CNN model presented in this paper, this can be viewed as different variants of proposed method. We tested two kinds of low level features: histogram of oriented gradients (HOG) and local binary pattern (LBP) (DET + HOG and DET + LBP). The ROC curves are shown in Figure 9, and the classification accuracies are listed in Table 3. From the results, we can see the proposed CNN model outperforms the two variants with the HOG and LBP by a large margin.

#### 5.2.3. Comparison with Previous Work

We also compare the performance of our method with two previous methods [13,14]. These two methods perform image classification on the liver parenchyma which corresponds to the tissues under the liver capsules in our data. We select ROIs under the liver capsules and use the two method to classify the selected images. The results are given in Figure 9 and Table 3. Our method performs better than the two previous methods in the classification accuracy and the AUC, which proves the effectiveness of our framework.

#### 5.2.4. Impact of Patch Size

**Figure 10:**
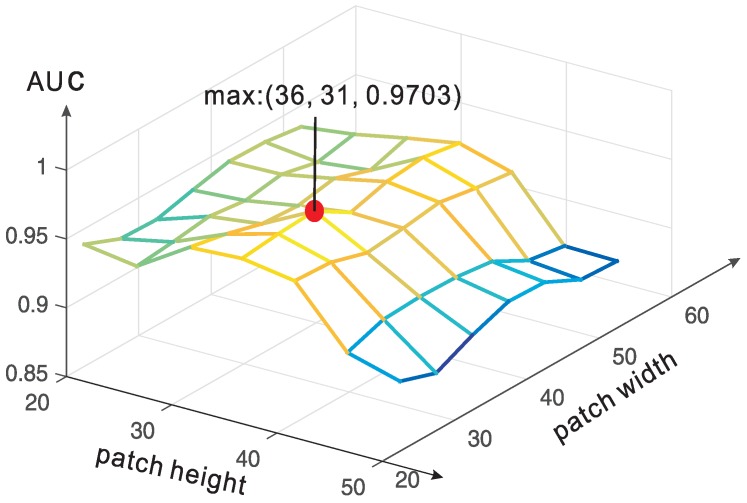
The performance of proposed method with different patch sizes.

In order to understand of how patch size influences the performance, we evaluate the performance with different patch sizes, and the results are reported in Figure 10. We have obtained the highest AUC of 0.9703 with patch size: (36,31). In the experiments, We use a patch size of (40,40) instead of the optimum one, since this optimum size is obtained with the help of the labels in testing data. From Figure 10, we learn that with the CNN model, patch size of 30–40 can generally yield high performance.

## 6. Conclusions

We propose in this paper a method for automatic diagnosis of cirrhosis with ultrasound images. With the guidance of an effective liver capsule extractor, the automatic diagnosis task can be effectively accomplished through image classification with a transferred deep classification model. Systematic experimental results have demonstrated the effectiveness of proposed method which can be implemented for target treatment to minimize fatalities.

## Figures and Tables

**Table 1:** The statistics of the subjects for evaluation.

Type	Total	Male	Female	Age
normal	44	20	24	48.8 ± 16.2
cirrhosis A	18	10	8	51.4 ± 10.5
cirrhosis B	16	6	10	50.3 ± 11.2
cirrhosis C	13	7	6	55.5 ± 11.3
cirrhosis total	47	23	24	52.2 ± 10.9

**Table 2:** Evaluation results of proposed liver capsule detection method.

	Total	Success	Percentage	Mean Completeness
normal	44	44	100%	0.99
diseased	47	38	81%	0.78
overall	91	82	90%	0.88

**Table 3:** The classification accuracies in 3-fold cross validation and the AUCs (area under roc curve).

	DET + HOG	DET + LBP	DET + CNN	GT + CNN	[13]	[14]
Accuracy (1)	0.806	0.871	0.968	0.968	0.839	0.871
Accuracy (2)	0.839	0.742	0.742	0.742	0.677	0.71
Accuracy (3)	0.862	0.828	0.897	0.966	0.828	0.828
mean	0.836	0.814	0.869	0.892	0.781	0.803
AUC	0.921	0.881	0.951	0.968	0.875	0.836
